# The Versatility of NADES Across Applications

**DOI:** 10.3390/molecules30193862

**Published:** 2025-09-24

**Authors:** David S. Freitas, Artur Ribeiro, Artur Cavaco-Paulo, Carla Silva

**Affiliations:** 1 Centre of Biological Engineering, University of Minho, Campus de Gualtar, 4710-057 Braga, Portugal; davidsfreitas@ceb.uminho.pt (D.S.F.); arturibeiro@ceb.uminho.pt (A.R.); artur@deb.uminho.pt (A.C.-P.); 2 LABBELS—Associate Laboratory, 4710-057 Braga, Portugal

**Keywords:** deep eutectic solvents, sustainable solvents, enzymatic processes, extraction, Pharma-cosmetics

## Abstract

Natural deep eutectic solvents (NADES) are produced by combining natural compounds, such as sugars, amino acids, or organic acids, to form a liquid at room temperature. Compared to other solvents, NADES own several strengths, including cost-effectiveness, ease of preparation, tunable properties, biorenewability, and biodegradability, making them suitable for a wide range of industrial sectors. Research on NADES requires careful consideration of their composition and physicochemical properties, as these can significantly influence their range of applications. In this context, the main objective of this review is to provide insights into the application of NADES in different areas that go from enzymatic processes and extraction of bioactives to the formulation of pharmaceutical and cosmetic products. This review includes several case studies on the use of enzyme–NADES systems (lipase and laccase) to synthesize new materials and on the extraction of bioactives with NADES, highlighting their direct application in cosmetics and pharmaceutical formulations.

## 1. Introduction

The growing demand for environmentally sustainable alternatives to conventional solvents has spurred the development of deep eutectic solvents (DES), a class of compounds regarded as a green evolution of ionic liquids (IL). DES combine several advantageous properties, including low toxicity, low cost, biodegradability, and high thermal and chemical stability, which make them attractive for applications in green chemistry, electrochemistry, and biotechnology [1,2,3,4,5].

A particularly promising subclass of DES is the natural deep eutectic solvents (NADES), composed exclusively of naturally derived constituents such as sugars, amino acids, organic acids, and choline derivatives [1]. These solvents are formed through a eutectic mixture of a hydrogen bond donor (HBD) and a hydrogen bond acceptor (HBA), producing a highly stable and tunable hydrogen-bonding network. Beyond their intrinsic biodegradability, NADES are also non-flammable, exhibit negligible volatility, and demonstrate excellent biocompatibility. Their physicochemical properties can be precisely tailored by varying the molar ratios of their components, while the addition of small amounts of water or other benign co-solvents further modulates viscosity, polarity, and conductivity, expanding their applicability [1,2,3,4,5].

Compared to IL, DES, and traditional organic solvents, NADES are significantly more biodegradable and less toxic, strongly aligning with the principles of green chemistry [1]. Among their most valuable features is their exceptional solubilizing capacity for a wide range of compounds, including those with low water solubility. This capacity arises from the extensive intermolecular hydrogen bonding within the solvent matrix, which facilitates the dissolution of both natural products and synthetic molecules [1,6,7,8,9,10]. Their ability to mimic intracellular solvents further enhances their relevance for biological and pharmaceutical applications.

For bioactive extraction, NADES not only improve solubility but also enhance the stability of sensitive molecules such as polyphenols, alkaloids, and flavonoids, reducing degradation during processing [4,11,12,13]. In enzymatic reactions, NADES provide a favorable microenvironment that can increase enzyme activity, selectivity, and stability while minimizing denaturation, making them excellent media for biocatalysis. In cosmetics, their natural origin, low toxicity, and skin-friendly profile enable safe formulations, while their ability to solubilize both hydrophilic and hydrophobic compounds allows the incorporation of a broader range of active ingredients. Consequently, NADES have gained significant attention in natural product extraction, biocatalysis, and cosmetic applications, where efficient solubilization, biocompatibility, and eco-safety are essential [1,14,15]. Their versatility and sustainability position NADES as a promising platform in the transition toward greener industrial processes.

Table 1 contains several NADES selected as case studies in this review, with a focus on their roles in enzymatic synthesis and bioactive compound extraction.

## 2. The Use of NADES on Enzymatic Processes

Enzymatic processes involve biological reactions catalyzed by enzymes and offer several advantages over conventional chemical methods, including high specificity, the ability to function under mild conditions, and the capacity to catalyze complex transformations [14,19,20]. These attributes have led to widespread application of enzymatic processes across diverse industries, such as pharmaceuticals, food and beverages, and biofuels. As a result, there has been growing interest in identifying environmentally friendly and effective solvents that can support and enhance enzymatic performance. In this context, NADES have emerged as promising candidates.

NADES offer a range of advantages in biocatalysis, including enzyme stabilization, enhanced catalytic activity, and improved substrate solubility and selectivity [15,21,22]. Their ability to create a stable and protective microenvironment helps mitigate enzyme denaturation and degradation, resulting in higher catalytic efficiency under operational conditions [15]. These properties have enabled the successful integration of NADES into a variety of enzymatic processes. Reactive NADES have been effectively used in lipase-catalyzed esterification, particularly in the synthesis of polyol- and carbohydrate-based biosurfactants, where they enhance both activity and thermal stability [23]. In oxidative biocatalysis, choline chloride-based NADES have functioned as both reaction media and sacrificial electron donors in peroxygenase-catalyzed reactions, supporting enzyme stability and enabling in situ hydrogen peroxide generation [24]. In biomass processing, NADES pre-treatment has significantly improved the enzymatic hydrolysis of lignocellulosic biomass, a key step in biofuel production [25]. Additionally, NADES have been explored as electrolytes in electro-enzymatic systems, facilitating efficient CO_2_ reduction and NADH regeneration [26]. Recent studies further highlight the importance of developing sustainable enzymatic platforms. For example, novel epilactose- and tagatose-producing enzymes have been applied to expired milk and whey, converting waste substrates into high-value functional sugars with excellent purity and recovery [27,28]. Similarly, enzymatic enrichment of milk with sialyloligosaccharides demonstrates the potential of tailored biocatalysis to enhance nutritional value [29]. The integration of NADES into these processes could further improve outcomes by providing a stabilizing microenvironment for sensitive enzymes, enhancing substrate and product solubility, and protecting bioactive compounds from degradation. This combination not only increases enzymatic efficiency but also aligns with green chemistry principles, enabling sustainable, low-toxicity, and high-value transformations in food and bioprocessing applications. These examples demonstrate the versatility of NADES and their potential to enhance enzymatic processes in green and sustainable industrial applications [21,22,23,24,25,26,27,28,29,30,31,32].

### 2.1. The Role of NADES on Lipase Stability and Reactivity

Lipases are a class of hydrolase enzymes that catalyze the hydrolysis of triglycerides into free fatty acids and glycerol [33]. This reaction plays a critical role in lipid digestion and absorption, with pancreatic lipase being a key enzyme secreted into the small intestine to facilitate the breakdown of dietary fats. In addition to their physiological functions, lipases exhibit remarkable catalytic versatility, enabling them to operate in both aqueous and non-aqueous environments. This flexibility has positioned lipases as valuable biocatalysts in a variety of industrial applications, particularly in synthetic reactions such as esterification, transesterification, and interesterification [34]. These transformations are essential in the production of biodiesel, flavor esters, and structured lipids. Furthermore, lipases are utilized in a wide array of consumer and industrial products, including detergents, cosmetics, textiles, leather, and paper [35]. Their broad substrate range, operational stability under mild conditions, and alignment with green chemistry principles underscore their growing importance as sustainable biocatalysts across diverse sectors.

NADES have recently emerged as promising green solvents that significantly enhance the activity and stability of lipases, making them attractive alternatives to conventional organic solvents in biocatalysis [36,37]. Several studies have demonstrated that NADES composed of choline chloride in combination with hydrogen bond donors such as glycerol, urea, or sugars significantly improve the activity, selectivity, and thermal stability of lipases under challenging reaction conditions. For instance, choline chloride-based systems have been successfully used in the esterification of fatty acids with polyols to produce sugar-based biosurfactants and biodegradable polyesters [36,38]. In another example, NADES compositions, such as choline chloride combined with sugars or glycerol, can substantially improve both the activity and catalytic efficiency of lipases, including *Candida antarctica* lipase B and porcine pancreatic lipase. These solvent systems not only accelerate reaction rates but also extend the operational stability of lipases under conditions that typically impair enzymatic function, such as elevated temperatures and extreme pH values [31,36,37]. For example, *Rhizopus niveus* lipase immobilized on nanodiamonds exhibited enhanced thermal stability and sustained catalytic activity when employed in NADES media. Similarly, a NADES composed of betaine and urea has been shown to preserve lipase activity over prolonged reaction periods. Beyond their stabilizing effects, NADES offer remarkable versatility; they may serve either as primary solvents or as co-solvents in aqueous mixtures, thereby facilitating the dissolution of both hydrophobic and hydrophilic substrates. This attribute is particularly advantageous in biocatalytic transformations involving water-soluble reactants, which often pose solubility challenges in conventional organic solvents [39].

NADES interact with lipase through hydrogen-bonding networks that stabilize the tertiary structure, while the tunable polarity of NADES improves substrate accessibility and reduces aggregation or denaturation. Reduced water activity in NADES can limit hydrolytic side reactions, and specific NADES components may interact with residues near the active site, modulating flexibility and enhancing catalytic efficiency [23,40].

Reactive NADES have been effectively used in lipase-catalyzed esterification, particularly in the synthesis of polyol- and carbohydrate-based biosurfactants and biodegradable polyesters [23]. In these systems, NADES increase enzyme stability, thermal tolerance, and operational lifetime. Compared with traditional organic solvents, lipases in NADES exhibit higher conversion rates, improved regio- and enantioselectivity, and prolonged activity over multiple reaction cycles. The hydrogen-bond donors and acceptors in NADES stabilize the catalytic triad and critical surface residues, preserving the active conformation and facilitating substrate binding [23].

Additionally, the versatility of NADES allows them to function as either primary solvents or co-solvents in aqueous mixtures. Their ability to solubilize both hydrophilic and hydrophobic substrates enhances reaction efficiency, particularly for polar polyols and sugars that are poorly soluble in conventional organic solvents. By modulating the local microenvironment polarity and maintaining enzyme flexibility, NADES can further improve catalytic efficiency and product selectivity, highlighting their unique functional role in lipase-catalyzed transformations [41].

Overall, the application of NADES in lipase-catalyzed reactions represents a sustainable and multifunctional approach: they act simultaneously as enzyme stabilizers, microenvironment modulators, substrate solubilizers, and green reaction media. These properties collectively enable higher activity, selectivity, and stability than conventional solvents, making NADES a superior choice for lipase-driven biocatalysis in industrial applications [42].

#### Lipase–NADES Systems for the Synthesis of Biopolyesters: A Case Study

**Figure 1:**
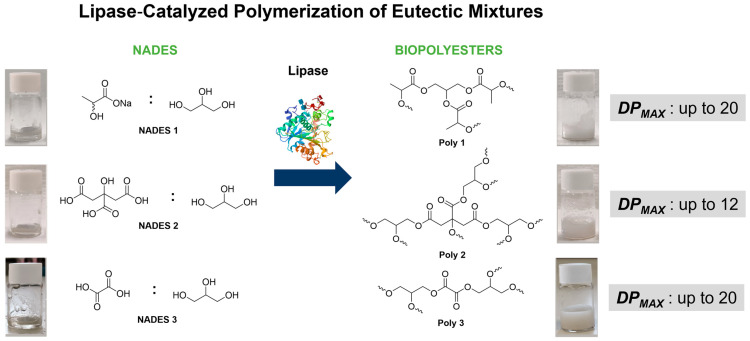
Enzymatic synthesis of three polyesters using lipase, with NADES (1, 2, and 3) serving as both reagents and solvents: the initial and final pictures of the solution reactions and the maximum degrees of polymerization (*DP_max_*) of the polyesters obtained (figure adapted from [17]).

A novel approach to polyester synthesis has been developed, using NADES components as a sustainable source of monomers. This method employs lipase-catalyzed esterification in an anhydrous environment, resulting in a greener and more efficient polyester production (Figure 1). Three NADES containing glycerol and sodium lactate (NADES 1 (1:1 ratio)), citric acid (NADES 2 (1:1 ratio)), or oxalic acid (NADES 3 (3:1 ratio)) were used to produce polyesters via polymerization reactions catalyzed by lipase from *Aspergillus oryzae*. For lipase-catalyzed polymerization (esterification), the NADES were lyophilized to prevent reverse catalysis reactions (hydrolysis). The polymerization of NADES catalyzed by lipase results in the formation of reaction water, which reduces the viscosity of the reaction medium. When the viscosity plateau was reached (≈4 h), it confirmed the existence of an equilibrium between esterification and the reverse hydrolysis reaction promoted by lipase. This study used magnetic, ultrasonic, and mechanical agitation to ensure polymerization of NADES constituents in the presence of lipase. Ultrasound was found to be the least effective for producing the intended polyesters. The mechanical agitation method emerged as particularly promising for industrial applications, showing a high degree of polymerization (DP) in the synthesized polyesters. This study showcased impressive polyester conversion rates, exceeding 70%, with a minimum of 20 monomeric units (Figure 1). These results emphasize the importance of striking the right balance between enzymatic and acid catalysis in NADES-based polyester production, offering a greener and more environmentally friendly approach to polyester synthesis [17].

### 2.2. The Role of NADES on Laccase Stability and Reactivity

Laccases (EC 1.10.3.2) are multicopper oxidases widely distributed across fungi, plants, bacteria, and insects. These enzymes catalyze the oxidation of a broad range of substrates, including phenolic and non-phenolic compounds, while reducing molecular oxygen to water. In nature, laccases participate in essential processes such as lignin degradation, morphogenesis, stress response, metal ion homeostasis, and detoxification of antimicrobial agents [43,44]. Their ability to oxidize compounds like catechol, catechin, gallic acid, ferulic acid, syringaldehyde, vanillin, and coniferyl alcohol under mild conditions, using only oxygen as the oxidant, positions laccases as attractive tools for green chemistry. They have shown particular promise in industrial sectors such as textiles, wastewater treatment, food processing, pharmaceuticals, and polymer synthesis. Despite their broad applicability, laccases face challenges related to stability and catalytic efficiency under industrial conditions, where fluctuations in temperature, pH, and solvent composition can significantly impair activity. This has led to the exploration of novel solvent systems to enhance enzyme performance and operational resilience [43,45].

NADES have emerged as a promising class of alternative solvents for biocatalysis. Comprising combinations of naturally occurring compounds—such as sugars, amino acids, organic acids, and quaternary ammonium salts—NADES are characterized by low toxicity, biodegradability, and the ability to form extensive hydrogen bonding networks. The physicochemical properties of NADES—namely viscosity, polarity, hydrophobicity, and hydrogen bonding capacity—are tunable based on constituent selection and molar ratios. These features offer significant advantages for stabilizing enzymatic structures and enhancing catalytic turnover [16,22,46]. NADES influence laccase properties through several pathways: (i) hydrogen-bonding and ionic interactions with surface residues stabilize the enzyme’s tertiary structure; (ii) the polarity of the NADES microenvironment improves substrate accessibility to the active site; (iii) reduced water activity minimizes hydrolytic degradation and denaturation; and (iv) controlled viscosity balances mass transfer and enzyme stabilization, optimizing catalytic efficiency [22,46,47].

Multiple studies have demonstrated that laccase activity and stability are highly dependent on the specific composition of the NADES. For instance, Khodaverdian et al. [22] found that a 2:1 molar ratio of glycerol to betaine significantly improved both laccase activity and thermal stability compared to aqueous mixtures containing the same components. This protective effect arises from NADES’ ability to maintain enzyme conformation and prevent thermal unfolding, a stabilization not achievable with conventional organic solvents [16,46]. Similarly, NADES based on choline chloride and hydroxyl-rich donors (e.g., glycerol, sugars) have been reported to enhance catalytic efficiency. Chloride ions and hydroxyl groups in NADES support substrate binding, maintain the redox state of copper centers in laccase, and preserve enzymatic activity under operational conditions [20,44].

The viscosity of NADES can hinder substrate diffusion and enzyme–substrate interactions. The partial addition of water or buffering agents has been shown to mitigate this issue by reducing viscosity and improving mass transfer, without significantly disrupting the hydrogen bond network that stabilizes the enzyme. This approach has demonstrated enhanced catalytic efficiency of laccase in NADES-based media and is regarded as a key strategy for industrial scale-up [16,44,48]. For example, Khlupova et al. [48] employed *Trametes hirsuta* laccase to polymerize dihydroquercetin in a 60% (*v*/*v*) DES–buffer system (betaine:glycerol, 1:2 molar ratio), achieving both high enzymatic activity and stability under operational conditions. Laccase-catalyzed oxidative polymerization of flavonoids and other phenolic compounds in NADES environments has opened new avenues in bio-based polymer production. Ünlü et al. [49] demonstrated successful synthesis of polycatechins using NADES composed of choline chloride:glycerol and betaine:mannose. These systems enabled solvent substitution without compromising polymer yield or enzyme efficiency, highlighting their potential in sustainable material development [49].

The integration of laccases with NADES-based solvent systems represents a significant advancement in biocatalysis, particularly for industrial applications requiring enzyme resilience and environmental compatibility. The tunable properties of NADES allow for the creation of enzyme-friendly environments that promote both activity and stability, addressing key limitations of traditional aqueous or organic solvent systems. Future research should focus on the systematic design of NADES formulations tailored to specific substrates and reaction conditions, as well as on enzyme engineering and immobilization strategies to further enhance performance [16,22,46]. With continued development, laccase–NADES systems have the potential to become central to the next generation of sustainable biotechnological processes.

#### Laccase–NADES Systems for the Synthesis of New Polymers: A Case Study

**Figure 2:**
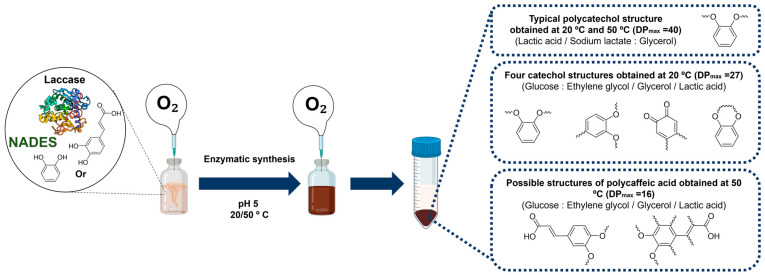
Reactional scheme for the enzymatic synthesis of catechol or caffeic acid-based polyphenolic compounds using a laccase-NADES system (figure adapted from [16]).

In a recent study, citric acid, oxalic acid, lactic acid, glucose, glycerol, ethylene glycol, and sodium lactate were used as raw components to produce NADES 1, 2, and 4–13 for the laccase-assisted polymerization of catechol and coffee phenols (Figure 2). The study found that laccase stability decreased with longer exposure times, higher temperatures, and increasing NADES concentrations. Among the tested solvents, the NADES composed of glucose and alcohol (NADES 8) best preserved enzyme activity, likely due to the stabilizing properties of sugars and alcohols. The laccase conformational structure remained virtually unchanged in the presence of the studied NADES, as confirmed by molecular modeling and intrinsic fluorescence data. The optimal reaction conditions for laccase-catalyzed polymerization were established by balancing enzyme stability and activity, with reactions conducted at 20 °C and 50 °C in a 10% (*w*/*w*) NADES solution in 0.1 M sodium acetate buffer at pH 5. It was also found that the introduction of supplemental oxygen further increased the conversion rates under these conditions. In this study one found that employing NADES with organic acid, a base, and alcohol resulted in a singular type of polycatechol at 20 °C. However, when oxidation reactions were conducted in acetate buffer or NADES containing glucose, four distinct polycatechol-derived structures emerged, specifically in the presence of glycerol, ethylene glycol, and DL-lactic acid. At 50 °C, catechol polymerization typically produces a single product. The results evidenced that when NADES were used as the reaction medium, catechol and caffeic acid exhibited a higher affinity for contact with laccase compared to when acetate buffer was used. These findings highlight the emerging role of NADES as an alternative green solvent in the synthesis of innovative polyphenolic materials [16].

## 3. The Use of NADES in the Extraction Processes

The extraction of bioactive compounds from natural sources, particularly plants, is a fundamental step in the development of pharmaceuticals, nutraceuticals, and functional foods. This process involves isolating target compounds from complex biological matrices using a variety of techniques, ranging from conventional methods such as maceration and decoction to advanced technologies like ultrasound- and microwave-assisted extraction. Modern extraction approaches offer significant advantages, including higher yields, reduced processing times, and lower solvent consumption [50,51]. Additionally, they support the valorization of agro-industrial by-products, contributing to sustainability and the circular economy. However, several challenges persist, such as maintaining the structural integrity and bioactivity of sensitive compounds, overcoming the complexity of plant matrices, and addressing issues of scalability and cost. Traditional methods also raise environmental concerns due to energy demands and solvent waste [50,51]. As such, future directions in extraction science increasingly emphasize the adoption of green and bio-inspired technologies, the use of eco-friendly solvents like NADES, and the optimization of process parameters to enhance selectivity, efficiency, and environmental compatibility. In this regard, NADES are becoming increasingly popular as environmentally friendly solvents due to their extraction capabilities, simple composition, non-volatile nature, low energy consumption, biodegradability, and low toxicity [52,53]. Their composition—typically based on natural metabolites such as sugars, organic acids, amino acids, and choline derivatives—enables them to disintegrate without generating harmful by-products, aligning well with the principles of green chemistry. NADES can be effectively employed to extract bioactive compounds due to their high thermal stability and exceptional solvation capacity for a broad spectrum of polar and non-polar molecules. Their capacity to form extensive hydrogen bonding networks with analytes such as phenolics, pigments, carotenoids, and flavonoids is a key factor in their extraction efficiency [11,12,52,53,54,55].

A major advantage of NADES lies in their customizable properties: by varying the types and ratios of the hydrogen bond donor and acceptor components, researchers can tailor NADES to match the physicochemical properties of specific target compounds. Their low volatility and non-flammability also enhance safety and sustainability during both lab-scale and industrial operations. Furthermore, NADES are compatible with food, pharmaceutical, and cosmetic matrices due to their natural origin, potentially allowing for direct use of the extract without the need for solvent removal, which is a significant advantage over traditional organic solvents [3,4,5]. In addition, NADES have demonstrated synergistic effects when combined with other green extraction technologies, such as ultrasound-assisted extraction and microwave-assisted extraction, further improving yield, reducing extraction times, and decreasing solvent volumes [55,56]. Studies investigating the influence of multiple variables—such as solvent composition, polarity, viscosity, temperature, and extraction time—are essential for optimizing these processes. For example, a recent comparative study demonstrated that a NADES composed of lactic acid and levulinic acid outperformed both ionic liquids and conventional solvents in extracting 20-hydroxyecdysone from spinach [57]. However, despite their advantages, NADES also present certain limitations. One of the most significant challenges is their inherently high viscosity, which can markedly hinder mass transfer and reduce diffusion rates, particularly at room temperature. To overcome this, external energy inputs—such as heating or agitation—are often required. Alternatively, dilution with water or ethanol may be employed, though this can compromise the solvent’s selectivity and solubility characteristics. [56]. Another significant drawback is the difficulty of separating target compounds from the NADES matrix due to their low volatility. This complicates downstream processing and solvent recovery. Although methods such as liquid–liquid extraction, antisolvent precipitation, and steam distillation using auxiliary solvents like water, ethanol, and ethyl acetate have been proposed, they introduce additional steps, costs, and environmental burdens [3,12].

Furthermore, the lack of standardization in NADES formulations and extraction protocols limits reproducibility and scalability. The vast number of possible combinations of components means that optimized formulations are often highly specific and not broadly applicable [58]. While NADES are generally regarded as safe, the cytotoxicity of some mixtures, particularly those containing organic acids or in high concentrations, must be evaluated for applications in the pharmaceutical, nutraceutical, and food industries. Toxicological data is still limited, and regulatory frameworks for NADES’ use in consumer products are underdeveloped [59,60].

Moreover, while promising at the laboratory scale, the translation of NADES-based extraction to industrial levels remains constrained by technical and economic barriers, including the cost of raw components, challenges in solvent recycling, and the need for process redesign to accommodate their unique physical characteristics [58].

NADES offer a sustainable and versatile alternative to conventional organic solvents for the extraction of natural compounds, especially when integrated with modern green extraction technologies. However, to fully realize their industrial potential, further research is needed—particularly in the areas of formulation optimization, toxicity assessment, process integration, and economic feasibility.

### Extraction of Bioactive Compounds Using NADES as Solvents: A Case Study

**Figure 3:**
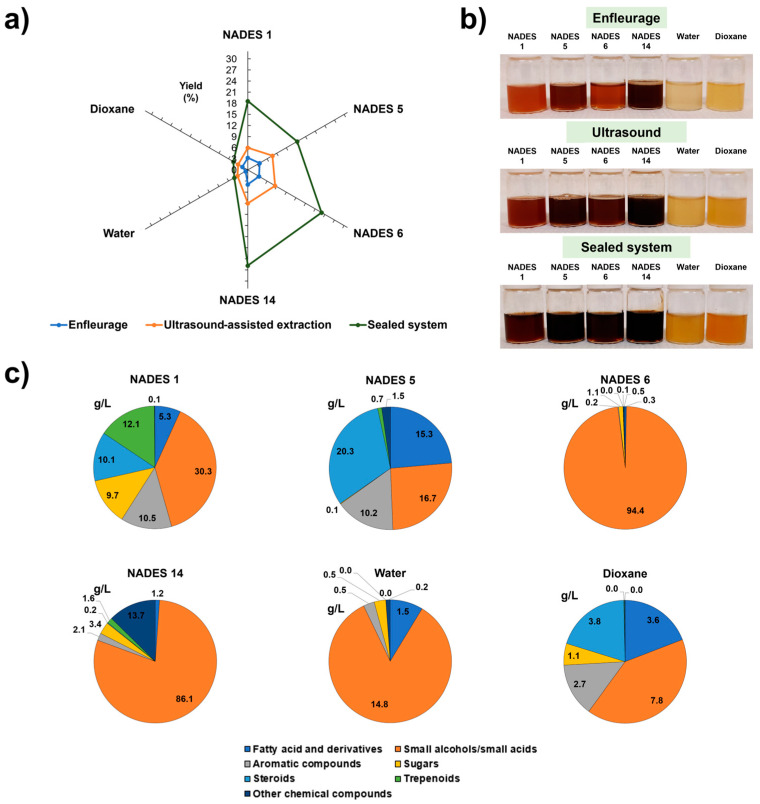
Extraction yield using NADES as solvents and different extraction methods (**a**) and the NADES-extracts aspect after the extraction process (**b**). In (**c**), the extractive concentrations (g/L) obtained from cork are represented using a sealed extraction system with NADES (1, 5, 6, and 14), water, and 1,4-dioxane as extraction solvents. Note: Enfleurage method: cork 0.7 g; solvent 15 mL; room temperature; 3 days; 3 cycles; Ultrasound-assisted: cork 0.7 g; solvent 10 mL; 50 °C; 6 h; 3 cycles; Sealed system: cork 0.7 g; solvent 5 mL; 100 °C 6 h; 3 cycles (adapted from [13]).

Recently, NADES’ improved capacity to extract bioactive compounds from *Quercus suber* cork was demonstrated when compared to harmful methods (Figure 3). NADES 1 (GLY:SL), 5 (LA:GLY), 6 (EG:SL), and 14 (LA:SC) yielded higher extraction yields than dioxane, influenced by pH, polarity, viscosity, and density, as well as the extraction method (Figure 3a). NADES yielded three to five times higher than control solvents like water and dioxane. The sealed system showed the highest extraction capacity, surpassing ultrasound by a factor of two and enfleurage by three. The closed system yields were either equal to or greater than those from previously reported methods that require more hazardous solvents. NADES 5 and 14, formulated with lactic acid, appear to facilitate an acid-catalyzed extraction mechanism, resulting in the efficient recovery of high-value bioactive compounds, including fatty acids and their derivatives, aromatic compounds, and terpenoids. Conversely, NADES 1 and 6 promote a base-catalyzed environment, leading to significant degradation of cork biopolymers and subsequent extraction of their monomeric constituents. Overall, the results indicate that acidic NADES (LA:GLY and LA:SC) extract a wide range of cork extractives, while basic NADES (EG:SL and GLY:SL) extract primarily monomers from biopolymers present in cork. This study demonstrated the possibility of adjusting and developing new NADES for extracting bioactives from cork, focusing only on the compounds of interest [13].

A subsequent study proposed the utilization of NADES extracts derived from cork in cosmetic formulations without purification, showcasing heightened antioxidant activity and demonstrating no toxic effects on keratinocytes. Additionally, these extracts were found to be suitable as dyeing agents for coloring cotton fabrics [61].

## 4. The Use of NADES in Cosmetics

The development and application of NADES for extracting bioactive compounds from plants and food waste for cosmetic formulations is an expanding field of research and innovation [62]. Recently, NADES have been specifically designed with components compatible for use in personal care products, aligning with the increasing demand for natural, eco-friendly ingredients. These solvents enhance the solubility and stability of bioactive compounds, offering a sustainable and non-toxic alternative to conventional organic solvents commonly used in the cosmetics industry. Their ability to form extensive hydrogen-bonding networks enables the gentle solvation of sensitive ingredients, such as vitamins, polyphenols, flavonoids, plant extracts, and essential oIL, without compromising their bioactivity [62,63,64].

Several studies have demonstrated the effectiveness of NADES in extracting high-value compounds with antioxidant, anti-aging, anti-inflammatory, and antimicrobial properties, which are key functionalities in modern skincare formulations. Moreover, NADES can improve the chemical stability of these bioactives, protect them from degradation, and enhance their bioavailability when applied topically. Their natural origin and biocompatibility also make them suitable for direct incorporation into formulations, particularly in products aimed at “clean label” or organic markets [62,65,66]. NADES mixtures of betaine, glycerol, and glucose enabled efficient extraction of epigallocatechin gallate from green tea for use in topical antioxidants [67]. Choline chloride:lactic acid NADES were used to extract polyphenols from tomato pomace for antioxidant creams with antifungal properties [65]. Similarly, choline chloride:formic acid solvents were effective in extracting naringenin from *Searsia tripartita*, demonstrating anti-aging activity through the inhibition of skin-degrading enzymes [68]. Glycerol-based NADES extracted isoquercetin from *Ginkgo biloba*, *Cinnamomum camphora*, and *Cryptomeria japonica* leaves, showing strong anti-tyrosinase and anti-elastase effects [69]. Other applications include skin-whitening emulsions from *Morus alba* root using urea–glycerol NADES, antioxidant-rich facial masks from *Ixora javanica*, and anti-inflammatory grape pomace extracts for creams, supporting NADES as multifunctional carriers and active ingredients [70,71].

NADES based on choline chloride combined with glycerol have also shown inhibitory activity against metalloproteases, including collagenase and elastase, which are implicated in skin aging. Their incorporation into cosmetic formulations not only enhances skin hydration perception but also facilitates uniform dispersion of actives across the skin surface, underscoring their potential as functional bioactive agents in anti-aging skincare [72].

From a sustainability perspective, NADES contribute to greener production processes by reducing the use of hazardous solvents and enabling the valorization of agro-industrial by-products, thus supporting circular economy principles. Despite challenges such as high viscosity, limited scalability, and the need for regulatory clarity, NADES are positioned to play an increasingly important role in the cosmetic industry. As consumer demand shifts toward safer, more sustainable formulations, NADES provide a promising platform for the development of next-generation, natural-based cosmetic products.

### NADES Extracts and Their Application in Cosmetics: A Case Study

**Figure 4:**
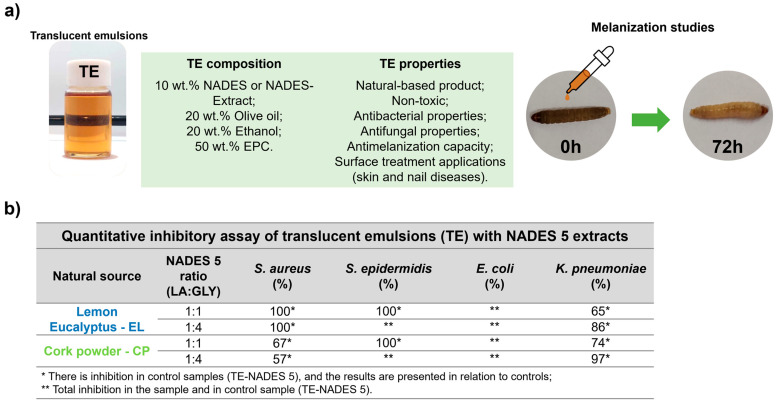
Scheme illustrating the use of NADES 5—LA:GLY (1:1 and 1:4) and their NADES extracts to produce translucent NADES-in-oil (TE) emulsions for cosmetic and pharmaceutical applications. The figure shows the composition, appearance, and properties of TEs and the improvement in the melanization state of larvae (*G. mellonella*) (right image) (**a**); and the antimicrobial and antifungal results of TEs with NADES–eucalyptus and NADES–cork extracts (**b**) of TE composed of NADES (control), NADES–eucalyptus (TE-EL), and NADES–cork (TE-CP) extracts (adapted from [18]).

A recent study addresses the use of NADES (pure NADES and NADES containing natural extract (NADES extract)) to create the first translucent NADES-in-oil emulsions (TEs) intended for cosmetic applications (Figure 4). The study selected NADES 5 (LA:GLY) in proportions of 1:1 and 1:4 due to its well-established characterization and physicochemical and structural aspects. NADES 5 allows for pH adjustment in the range of 1 to 8, maintaining its physical state. Its characteristics allow it to be easily incorporated into cosmetic or pharmaceutical formulations. NADES 5 was used to extract bioactive compounds from various plants, including *Lavandula augustifolia*, *Corymbia citriodora*, *Thymus vulgaris*, *Quercus suber*, *Allium sativum*, and *Origanum vulgare*. The extraction method provided high extraction yields and was easily scalable for industrial applications. The versatile application of NADES 5 demonstrated its effectiveness in extracting phenolic and flavonoid components, particularly its antioxidant and chelating properties. The preparation of TEs consisted of 50 wt.% egg-phosphatidylcholine (EPC), 20 wt.% ethanol, 20 wt.% olive oil, and 10 wt.% NADES 5, with or without extract (Figure 4a). All components used in the proposed method are inexpensive, easy to manufacture, and biocompatible. The study found that NADES with or without extract, as well as TE formulations, possess the ability to inhibit tyrosinase and elastase, enzymes linked to skin aging and loss of elasticity. All NADES 5 formulations, along with TE extracts, exhibited remarkable antifungal and antibacterial activity against various bacteria (Figure 4b). Their low toxicity was confirmed through testing on skin keratinocyte cell lines and larval models. Additionally, all TEs exhibited a significant improvement in larval melanization, indicating enhanced larval health (Figure 4a). Furthermore, these TEs demonstrated remarkable antimicrobial capacity, which, coupled with their low toxicity, enhances their potential for pharmaceutical and cosmetic applications [18].

Table 2 presents a summary of examples of NADES’ applications in the areas covered in this review.

## 5. Conclusions and Future Perspectives

**Figure 5:**
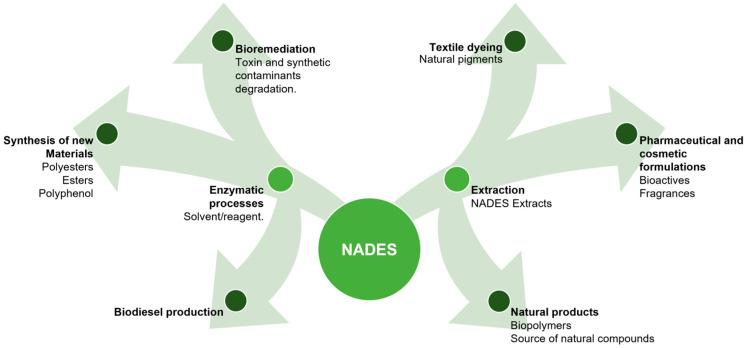
NADES’ applications across different sectors.

In today’s context, there is an open field for the application of NADES in enzymatic synthesis and extraction of bioactive compounds (Table 2 and Figure 5). These solvents can dissolve insoluble substances, including synthetic and natural substrates, enabling the production of novel products with high conversion rates. Research on NADES as green solvents/reagents for enzyme catalysis processes is expected to drive their widespread adoption across industries. Enzyme–NADES systems are also poised to play a pivotal role in bioremediation processes, aiding in the degradation of toxins and synthetic organic contaminants, thereby mitigating chemical pollution. Recent studies indicate that NADES can enable the synthesis of biodiesel through lipase-catalyzed reactions, offering immense promise for future industrialization due to its eco-friendliness and energy efficiency [32]. Understanding the behavior of enzyme–NADES systems is crucial for maximizing their utility in enzyme catalysis processes. This knowledge can drive innovation and sustainability across various domains. NADES have proven effective in extracting bioactive compounds from natural sources, but challenges remain in separating them from the solvent. Two approaches can be pursued: developing innovative separation technologies or directly incorporating NADES extracts into cosmetic and pharmaceutical formulations due to their low toxicity and high biodegradability.

Looking forward, natural pigments extracted using NADES offer promise for use in the textile industry, potentially reducing reliance on synthetic dyes. Additionally, NADES can be used for extracting biopolymers such as cellulose, chitin, lignin, and suberin, among others. These biomaterials are abundant sources of natural compounds and have significant applications in enzymatic catalysis processes and the pharmaceutical and cosmetic industries. With the scientific advancements delineated herein, it is anticipated that these technologies will be readily deployable in the industry in the foreseeable future.

## Figures and Tables

**Table 1:** Different NADES and their potential applications (their applications in different case studies are marked in green).

NADES	Components		Molar Ratio	Abbr.	Lipase-Assisted Catalysis	Laccase-Assisted Catalysis	Extraction Processes
1	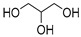 Glycerol	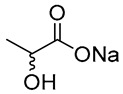 Sodium DL-lactate	1:1	GLY:SL [13,16,17]	Solvent and substrate	Solvent	Solvent
2	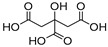 Citric acid	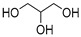 Glycerol	1:1	CA:GLY [16,17]	Solvent and substrate	Solvent	
3	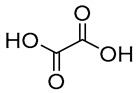 Oxalic acid	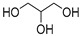 Glycerol	1:1	OA:GLY [17]	Solvent and substrate		
4	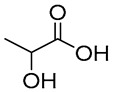 DL-Lactic acid	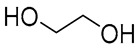 Ethylene glycol	1:1	LA:EG [16]		Solvent	
5	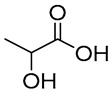 DL-Lactic acid	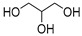 Glycerol	1:1; 1:4	LA:GLY [13,16,18]		Solvent	Solvent
6	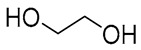 Ethylene glycol	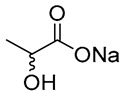 Sodium DL-lactate	1:1	EG:SL [13,16]		Solvent	Solvent
7	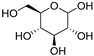 Glucose	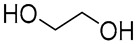 Ethylene glycol	1:1	GLU:EG [16]		Solvent	
8	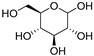 Glucose	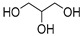 Glycerol	1:1	GLU:GLY [16]		Solvent	
9	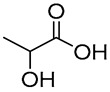 DL-Lactic acid	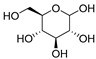 Glucose	1:1	LA:GLU [16]		Solvent	
10	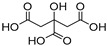 Citric acid	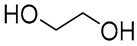 Ethylene glycol	1:1	CA:EG [16,17]		Solvent	
11	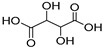 Tartaric acid	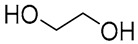 Ethylene glycol	1:1	TA:EG [16]		Solvent	
12	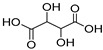 Tartaric acid	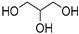 Glycerol	1:1	TA:GLY [16]		Solvent	
13	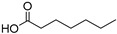 Enanthic acid	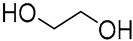 Ethylene glycol	1:1	EA:EG [16]		Solvent	
14	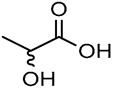 DL-Lactic acid (LA)	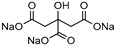 Sodium citrate (tribasic) (SC)	4:1	LA:SC [13]			Solvent

**Table 2:** Summary of NADES’ application: representative systems, target/substrates, main results, and role. Note: ↑ higher and ↓ lower.

Application Area	NADES System (Examples)	Target or Substrate	Key Outcomes	Role	References
Enzymes—Lipase	Choline Chloride:Glycerol, Choline Chloride:Sugar, Betaine:Urea, Sodium lactate:Glycerol, …	Fatty acids + polyols (esterification, biodiesel, biosurfactants), …	↑ Activity; ↑ Thermal/operational stability; ↑ Selectivity;	H-bonding stabilizes catalytic triad; tuned polarity improves substrate solubility; controlled water activity reduces hydrolysis; enables reusability.	[17,23,31,33,34,35,36,37,38,39,42]
Enzymes—Laccase	Betaine:Glycerol, Choline Chloride:Glycerol, Betaine:Mannose, Lactic acid:Glycerol, …	Phenolics, flavonoids, catechols (polymerization, oxidation), …	↑ Activity; ↑ Stability; ↑ Catalytic efficiency;	NADES stabilize tertiary folds and copper centers; polarity favors substrate access; water addition reduces viscosity while maintaining H-bond protection.	[16,20,22,43,44,45,48,49]
Extraction	Choline Chloride:Malic Acid, Choline Chloride:Glycerol/Urea, Lactic acid:Glycerol, Sugar-based NADES, …	Flavonoids, phenolics, polyphenols, alkaloids, fatty acids, terpenoids, …	↑ Yield; ↑ Selectivity; ↓ Toxic solvent use;	Strong H-bonding enhances solubility of polar compounds; tunable polarity allows selective extraction; biodegradable and safer than VOCs.	[3,4,5,11,12,13,50,51,52,53,54,55,56,57,58,59,60,61]
Cosmetics	Choline Chloride:Sorbitol, Choline Chloride:Glycerol, Urea:Glycerol, Lactic acid:Glycerol, …	Flavonoids, phenolics, polyphenols, alkaloids, fatty acids, terpenoids, …	↑ Solubility; ↑ Stability; ↑ Skin penetration, enzyme inhibition (anti-aging);	Biocompatible and eco-friendly; H-bonding stabilizes actives; enhances dermal delivery; NADES act as both extractants and formulation carriers.	[18,62,63,64,65,66,67,68,69,70,71,72]

## Data Availability

No new data were created or analyzed in this study. Data sharing is not applicable to this article.
